# Diagnostic accuracy of a prototype rapid chlamydia and gonorrhoea recombinase polymerase amplification assay: a multicentre cross-sectional preclinical evaluation^☆^

**DOI:** 10.1016/j.cmi.2018.06.003

**Published:** 2019-03

**Authors:** E.M. Harding-Esch, S.S. Fuller, S.-L.C. Chow, A.V. Nori, M.A. Harrison, M. Parker, O. Piepenburg, M.S. Forrest, D.G. Brooks, R. Patel, P.E. Hay, N. Fearnley, M.J. Pond, J.K. Dunbar, P.D. Butcher, T. Planche, C.M. Lowndes, S.T. Sadiq

**Affiliations:** 1) Applied Diagnostic Research & Evaluation Unit (ADREU), Institute for Infection & Immunity, St George's University of London, London, UK; 2) HIV/STI Department, National Infection Service, Public Health England, London, UK; 3) St George's University Hospitals NHS Foundation Trust, London, UK; 4) TwistDx Limited, Cambridge, UK; 5) Department of Sexual Health, University of Southampton, Southampton, UK; 6) Bradford Teaching Hospitals NHS Foundation Trust, Bradford, UK

**Keywords:** *Chlamydia trachomatis*, Diagnostic accuracy, *Neisseria gonorrhoeae*, Nucleic acid amplification tests, Performance evaluation, Point of care

## Abstract

**Objectives:**

Rapid and accurate sexually transmitted infection diagnosis can reduce onward transmission and improve treatment efficacy. We evaluated the accuracy of a 15-minute run-time recombinase polymerase amplification–based prototype point-of-care test (TwistDx) for *Chlamydia trachomatis* (CT) and *Neisseria gonorrhoeae* (NG).

**Methods:**

Prospective, multicentre study of symptomatic and asymptomatic patients attending three English sexual health clinics. Research samples provided were additional self-collected vulvovaginal swab (SCVS) (female participants) and first-catch urine (FCU) aliquot (female and male participants). Samples were processed blind to the comparator (routine clinic CT/NG nucleic acid amplification test (NAAT)) results. Discrepancies were resolved using Cepheid CT/NG GeneXpert.

**Results:**

Both recombinase polymerase amplification and routine clinic NAAT results were available for 392 male and 395 female participants. CT positivity was 8.9% (35/392) (male FCU), 7.3% (29/395) (female FCU) and 7.1% (28/395) (SCVS). Corresponding NG positivity was 3.1% (12/392), 0.8% (3/395) and 0.8% (3/395). Specificity and positive predictive values were 100% for all sample types and both organisms, except male CT FCU (99.7% specificity (95% confidence interval (CI) 98.4–100.0; 356/357), 97.1% positive predictive value (95% CI 84.7–99.9; 33/34)). For CT, sensitivity was ≥94.3% for FCU and SCVS. CT sensitivity for female FCU was higher (100%; 95% CI, 88.1–100; 29/29) than for SCVS (96.4%; 95% CI, 81.7–99.9; 27/28). NG sensitivity and negative predictive values were 100% in FCU (male and female).

**Conclusions:**

This prototype test has excellent performance characteristics, comparable to currently used NAATs, and fulfils several World Health Organization ASSURED criteria. Its rapidity without loss of performance suggests that once further developed and commercialized, this test could positively affect clinical practice and public health.

## Introduction

*Chlamydia trachomatis* (CT) and *Neisseria gonorrhoeae* (NG) are major contributors to the burden of sexually transmitted infections (STIs) in England and elsewhere [1], [2]. They are frequently asymptomatic (especially in women) [3], commonly remaining undiagnosed, and if untreated they can lead to serious complications [4], [5].

Currently, it can take up to 2 weeks to obtain CT and NG results and treatment after STI testing in sexual health clinics (SHCs) in the UK [6], but delays may be considerably longer in other settings [7]. During this period, sexual risk taking may continue, including acquisition of new partners [8]. Rapid and accurate CT/NG point-of-care tests (POCTs), enabling diagnosis and treatment of infected patients within the same clinical visit [9] (a ‘test and treat’ strategy), could potentially reduce rates of inappropriate presumptive treatment, shorten time to treatment, decrease rates of untreated CT and NG for patients lost to follow-up, limit onward transmission and reduce rates of sequelae [10], [11], [12].

British Association for Sexual Health and HIV guidelines state that CT and NG detection must use nucleic acid amplification tests (NAATs) (and/or culture for NG) [5], [13]. A number of rapid and point-of-care NAAT-based tests for CT and NG are being or have recently been developed [14]. Newer NAAT technologies that use isothermal amplification, avoiding the need for thermal cycling, have the potential to enable fast turnaround times from sample to result. TwistDx (Cambridge, UK) have developed an isothermal recombinase polymerase amplification (RPA) method, which can detect CT/NG infection (single NG target) in approximately 15 minutes, requires no thermal cycling, can be battery powered and has a reaction temperature of 37°C. The RPA CT/NG assay is run on an Alere i instrument (Alere, Waltham, MA, USA). The TwistDx RPA CT/NG assay is therefore an excellent candidate for development as a true molecular CT/NG POCT, allowing for test and treat pathways in SHCs, community and resource-poor settings [15].

We aimed to assess the diagnostic accuracy of the prototype TwistDx RPA assay for genital CT and NG detection on prospectively collected clinical samples from men and women in English SHCs.

## Methods

Ethical approval was granted by the London Bridge Research Ethics Committee (13/LO/0691). This report was written following Standards for Reporting Diagnostic Accuracy guidelines (Supplementary Table S1) [16].

### Sample size and recruitment

This prospective multicentre diagnostic accuracy evaluation was powered to obtain a minimum of 50 CT and 20 NG positive and 200 negative, samples for both male and female participants. Assuming 92% sensitivity and 99% specificity of the RPA CT/NG assay compared to standard NAATs, the 95% confidence intervals (CIs) obtained would be 81.2 to 96.8 and 96.4 to 99.9, respectively.

Assuming a CT prevalence of 8.3% (based on Genito-Urinary Medicine Clinical Activity Data set (GUMCAD) [17] from the South London SHC), 600 individuals would lead to the requisite number of CT-positive and -negative samples. With a lower expected NG prevalence of 3%, 800 participants were needed. In order to allow subgroup analysis by gender, we planned to recruit 400 men and 400 women.

### Study sites and participant selection

Three SHCs located in South London, Yorkshire and on the south coast of England participated. Eligible patients were recruited during routine consultations by clinic staff using the following eligibility criteria: age ≥16 years; attending the SHC; had not passed urine in the previous 2 hours; provided written informed consent for the collection of research samples; provided all sample types (men: first-catch urine (FCU) and meatal swabs before and after micturition; women: FCU and self-collected vulvovaginal swabs (SCVS)). Participant demographic and clinical data were collected on case report forms.

### Sample collection and processing

All samples for this evaluation were self-taken by participants after collection of routine clinical samples. A minimum volume of 20 mL FCU was collected from male subjects; an aliquot of 2 to 3 mL was taken for routine clinical testing, and the remainder was immediately stored at 2 to 8°C until shipment (twice weekly) on wet ice to TwistDx for RPA CT/NG testing. In addition, male participants were asked to self-collect two external penile meatal swabs, one before urination and the second after urination.

Women provided two SCVS, the first for the clinic's routine CT/NG NAAT, followed by an FCU specimen. FCU from female participants was processed as per FCU from male participants, except that no aliquots for routine CT/NG NAAT testing were taken. Research SCVS samples were eluted in clinic within 10 minutes of collection. Swabs were immersed and swirled in 1 mL lysis buffer for 5 seconds, left to stand in the lysis buffer for 90 seconds and then disposed of. Neutralization buffer (2 mL) was added to the lysis buffer and the tube inverted ten times. Tubes were stored at −20°C (or lower) before shipment (twice weekly) on dry ice to TwistDx for RPA CT/NG testing.

### Sample testing and resolution of discrepant results

Routine clinical NAAT testing was performed locally on male FCU and female SCVS samples, as per clinic standard practice (BD Viper CT/NG assay (Becton Dickinson, Oxford, UK) at the South London clinic; GenProbe Aptima CT/NG test (Hologic Gen-Probe, Marlborough, MA, USA) at the other clinics).

Research sample processing and testing were in accordance with TwistDx protocols, developed through internal optimization (unpublished data). The RPA CT/NG assay was performed on research samples (FCU for men, FCU and SCVS for women) by staff at TwistDx, who were unaware of the routine clinic NAAT and case report form results. FCU samples were processed through a size-exclusion chromatography device (Zeta Sep FPLC Desalting Columns; Generon, Slough, UK) for the purposes of desalting the sample before testing on the Alere i instrument.

The comparator test for male FCU samples was the routine clinic NAAT performed on male FCU; that for female SCVS and FCU was the routine clinic NAAT on female SCVS samples. Data were sent to the Applied Diagnostic Research & Evaluation Unit (ADREU), St George's University of London, where the routine clinic NAAT and RPA CT/NG assay results were compared for each participant and sample type. We defined the reference standard [16] as the routine clinic NAAT result when in agreement with the RPA CT/NG assay and no further testing was performed. Otherwise, all sample eluates from patients where a discrepant result had been found were tested at the TwistDx facility using the CT/NG GeneXpert as per manufacturer's instructions, with the sample type (FCU or swab) and initial CT or NG results masked. In these cases, the reference standard was defined as the resolved result when two out of three of the test results were in agreement.

### Data and statistical analysis

Data were entered into a database by ADREU. Participants for whom either the RPA CT/NG or routine clinic NAAT results were missing and/or who did not provide both sample types (swab and FCU) in the case of women, as per eligibility criteria, were excluded from analyses. Calculation of RPA CT/NG assay diagnostic accuracy measures (sensitivity, specificity, positive predictive value (PPV) and negative predictive value (NPV)) and their binomial exact 95% CIs was carried against the reference standard. Comparison of performance by subgroup (symptomatic vs. asymptomatic; female FCU vs. SCVS) was performed by the Pearson chi-square statistic. All analyses were conducted by Stata 12.0 software (StataCorp, College Station, TX, USA).

## Results

### Overview of participants

**Fig. 1:**
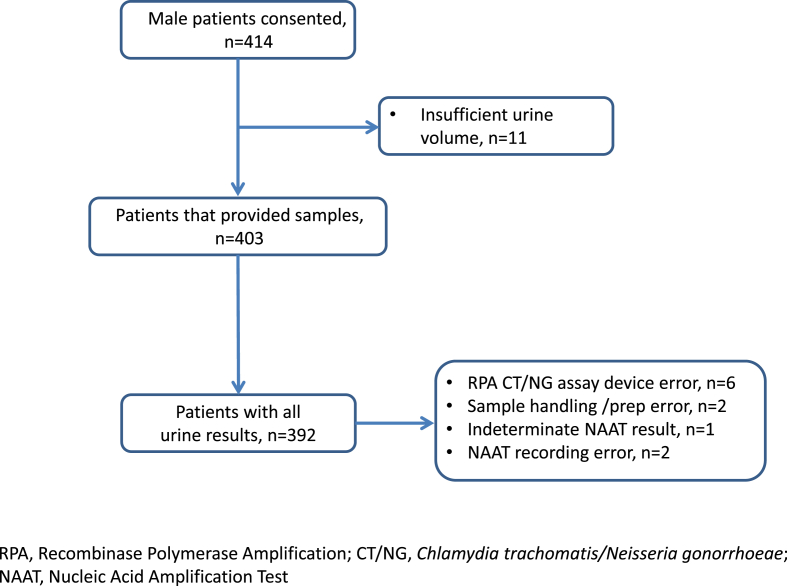
Patient and sample flow for male participants.

**Fig. 2:**
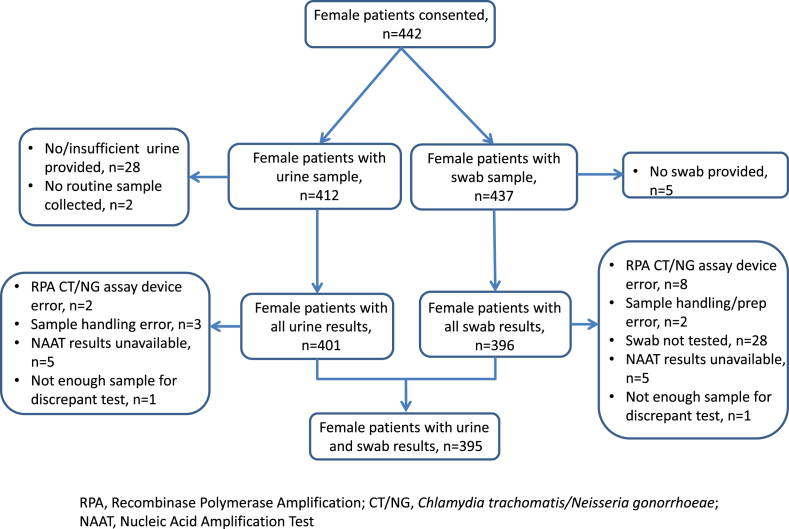
Patient and sample flow for female participants.

Recruitment took place May to September 2014. A total of 414 men and 442 women provided written informed consent (Fig. 1, Fig. 2). Both RPA CT/NG assay and routine clinic NAAT results were available for FCU for 392 (94.7%) of 414 men. A total of 395 (89.7%) of 442 women had both FCU and SCVS results available for all tests performed (RPA CT/NG for FCU and SCVS; routine clinic NAAT for SCVS). Participant characteristics are summarized in Table 1. Study CT positivity was 35 (8.9%) of 392 for male FCU, 29 (7.3%) of 395 for female FCU and 28 (7.1%) of 395 for SCVS. Corresponding NG positivities were 12 (3.1%) of 382, three (0.8%) of 395 and three (0.8%) of 395 (Table 2).

**Table 1:** Participant characteristics

Characteristic	Male participants			Female participants		
	Total	Positive for CT	Positive for NG	Total	Positive for CT	Positive for NG
Age						
16–19 years	15 (3.8)	1 (6.7)	0	40 (10.1)	5 (12.5)	0
20–24 years	126 (32.1)	22 (17.5)	6 (4.8)	157 (39.7)	18 (11.5)	0
25–34 years	148 (37.8)	11 (7.4)	2 (1.4)	140 (35.4)	4 (2.9)	1 (0.7)
35–44 years	64 (16.3)	0	3 (4.7)	41 (10.4)	1 (2.4)	1 (2.4)
45–64 years	37 (9.4)	1 (2.7)	1 (2.7)	16 (4.1)	0	0
65+ years	2 (0.5)	0	0	1 (0.3)	0	0
Clinic						
1	127 (32.4)	13 (10.2)	7 (5.5)	157 (39.7)	8 (5.1)	2 (1.3)
2	186 (47.4)	18 (9.7)	4 (2.2)	161 (40.8)	16 (9.9)	1 (0.6)
3	79 (20.2)	4 (5.1)	1 (1.3)	77 (19.5)	4 (5.2)	0
Contact						
No	340 (87.4)	18 (5.3)	10 (2.9)	363 (92.8)	16 (4.4)	2 (0.6)
CT only	33 (8.5)	14 (42.4)	2 (6.1)	22 (5.6)	11 (50.0)	0
NG only	7 (1.8)	2 (28.6)	0	1 (0.3)	0	1 (100)
Both CT and NG	9 (2.3)	1 (11.1)	0	5 (1.3)	0	0
Received CT/NG active medication since test/6 weeks before test						
No	375 (95.7)	34 (9.1)	11 (2.9)	367 (92.9)	27 (7.4)	3 (0.8)
Yes	17 (4.3)	1 (5.9)	1 (5.9)	28 (7.1)	1 (3.6)	0
Symptomatic						
No	249 (63.7)	18 (7.2)	2 (0.8)	208 (52.8)	14 (6.7)	1 (0.5)
Yes	142 (36.3)	16 (11.3)	10 (7.0)	186 (47.2)	14 (7.5)	2 (1.1)
Currently menstruating						
No				368 (93.4)	28 (7.6)	3 (0.8)
Yes				26 (6.6)	0	0

Data are presented as *n* (%). CT and NG positivity was defined as reference standard (positive by at least two of three tests: clinic NAAT, RPA CT/NG assay, Cepheid GeneXpert). Male participants were considered symptomatic if they reported one or more of: discharge (clear or cloudy liquid from penis); irritation at top of penis; itching; needing to pass urine more often than usual; pain/burning when urinating. Female participants were considered symptomatic if they reported one or more: itching; discharge (clear or cloudy liquid from vagina); pain/burning when urinating; needing to pass urine more frequently; pain during sex; bleeding after sex; bleeding in between periods; pelvic abdominal pain.

CT, *Chlamydia trachomatis;* NAAT, nucleic acid amplification test; NG, *Neisseria gonorrhoeae;* RPA, recombinase polymerase amplification.

**Table 2:** RPA CT/NG assay performance

Characteristic	Male participants		Female participants			
	FCU		FCU		SCVS	
	CT	NG	CT	NG	CT	NG
All participants						
No. positive/total	35/392	12/392	29/395	3/395	28/395	3/395
Positive	8.9%	3.1%	7.3%	0.8%	7.1%	0.8%
Sensitivity (%, 95% CI), *n/N*	94.3 (80.8–99.3), 33/35	100 (73.5–100), 12/12	100 (88.1–100), 29/29	100 (29.2–100), 3/3	96.4 (81.7–99.9), 27/28	66.7 (9.0–100), 2/3
Specificity (%, 95% CI), *n/N*	99.7 (98.4–100), 356/357	100 (99.0–100), 380/380	100 (99.0–100), 366/366	100 (99.1–100), 392/392	100 (99.0–100), 367/367	100 (99.1–100), 392/392
PPV (%, 95% CI), *n/N*	97.1 (84.7–99.9), 33/34	100 (73.5–100), 12/12	100 (88.1–100), 29/29	100 (29.2–100), 3/3	100 (87.2–100), 27/27	100 (15.8–100), 2/2
NPV (%, 95% CI), *n/N*	99.4 (98.0–99.9), 356/358	100 (99.0–100), 380/380	100 (99.0–100), 366/366	100 (99.1–100), 392/392	99.7 (98.5–100), 367/368	99.7 (98.6–100), 392/393
Symptomatic participants						
No. positive/total	16/142	10/142	14/186	2/186	14/186	2/186
Positive	11.3%	7.0%	7.5%	1.1%	7.5%	1.1%
Sensitivity (%, 95% CI), *n/N*	93.8 (69.8–99.8), 15/16	100 (69.2–100), 10/10	100 (76.8–100), 14/14	100 (15.8–100), 2/2	92.9 (66.1–99.8), 13/14	50 (1.3–98.7), 1/2
Specificity (%, 95% CI), *n/N*	99.2 (95.7–100), 125/126	100 (97.2–100), 132/132	100 (97.9–100), 172/172	100 (98.0–100), 184/184	100 (97.9–100), 172/172	100 (98.0–100), 184/184
PPV (%, 95% CI), *n/N*	93.8 (69.8–99.8), 15/16	100 (69.2–100), 10/10	100 (76.8–100), 14/14	100 (15.8–100), 2/2	100 (75.3–100), 13/13	100 (2.5–100), 1/1
NPV (%, 95% CI), *n/N*	99.2 (95.7–100), 125/126	100 (97.2–100), 132/132	100 (97.9–100), 172/172	100 (98.0–100), 184/184	99.4 (96.8–100), 172/173	99.5 (97.0–100), 184/185
Asymptomatic participants						
No. positive/total	18/249	2/249	15/208	1/208	14/208	1/208
Positive	7.2%	0.8%	7.2%	0.48%	6.7%	0.48%
Sensitivity (%, 95% CI), *n/N*	100 (81.5–100), 18/18	100 (15.8–100), 2/2	100 (78.2–100), 15/15	100 (2.5–100), 1/1	100 (76.8–100), 14/14	100 (2.5–100), 1/1
Specificity (%, 95% CI), *n/N*	100 (98.4–100), 231/231	100 (98.5–100), 247/247	100 (98.1–100), 193/193	100 (98.2–100), 207/207	100 (98.1–100), 194/194	100 (98.2–100), 207/207
PPV (%, 95% CI), *n/N*	100 (81.5–100), 18/18	100 (15.8–100), 2/2	100 (78.2–100), 15/15	100 (2.5–100), 1/1	100 (76.8–100), 14/14	100 (2.5–100), 1/1
NPV (%, 95% CI), *n/N*	100 (98.4–100), 231/231	100 (98.5–100), 247/247	100 (98.1–100), 193/193	100 (98.2–100), 207/207	100 (98.1–100), 194/194	100 (98.2–100), 207/207

CT and NG positivity was defined as reference standard (positive by at least two of three tests: clinic NAAT, RPA CT/NG assay, Cepheid GeneXpert). Male participants were considered symptomatic if they reported one or more of: discharge (clear or cloudy liquid from penis); irritation at top of penis; itching; needing to pass urine more often than usual; pain/burning when urinating. Female participants were considered symptomatic if they reported one or more: itching; discharge (clear or cloudy liquid from vagina); pain/burning when urinating; needing to pass urine more frequently; pain during sex; bleeding after sex; bleeding in between periods; pelvic abdominal pain.

CI, confidence interval; CT, *Chlamydia trachomatis;* FCU, first-catch urine; NAAT, nucleic acid amplification test; NG, *Neisseria gonorrhoeae;* NPV, negative predictive value; PPV, positive predictive value; RPA, recombinase polymerase amplification; SCVS, self-collected vulvovaginal swab.

### CT/NG RPA assay diagnostic accuracy

Table 2 summarizes the RPA CT/NG assay diagnostic accuracy estimates (Supplementary Table S2). In three (0.8%) of 392 FCU samples, RPA CT/NG results disagreed with routine clinic NAAT results for CT only (there were no NG discrepant results) (Supplementary Table S3A). After discrepant testing, zero of three RPA CT/NG results agreed with the resolved result. Subsequently, in men, all diagnostic accuracy measures were 100% for NG (12/12; 95% CI, 73.5–100 for sensitivity and PPV; 380/380; 95% CI, 99.0–100 for specificity and NPV). For CT, specificity and NPV were ≥99.4% (356/357; 95% CI, 98.4–100 and 356/358; 95% CI, 98.0–99.9, respectively), PPV was 97.1% (33/34; 95% CI, 84.7–99.9) and sensitivity was 94.3% (33/35; 95% CI, 80.8–99.3) (Table 2).

For women, 395 FCU and 395 SCVS were tested for CT and NG by the RPA CT/NG assay (Table 2). For CT, six (0.76%) of 790 (three FCU, three SCVS) results disagreed with the routine clinic NAAT SCVS result (Supplementary Table S3B). After discrepant testing, the RPA CT/NG assay agreed with the resolved result for all three FCU discrepant results and two of three SCVS discrepant results. For NG, seven (0.89%) of 790 (three FCU, four SCVS) RPA CT/NG results disagreed with the routine clinic NAAT SCVS result (Supplementary Table S3B). Of these, all three FCU and three of four SCVS discrepant results agreed with the resolved result. Thus, in women, all measures of diagnostic accuracy were 100% for FCU for both CT and NG. For CT and NG in SCVS, specificity and PPV were 100%, NPV was 99.7% (367/368; 95% CI, 98.5–100) and sensitivity was 96.4% (27/28; 95% CI, 81.7–100) for CT and 66.7% (2/3; 95% CI, 9.0–100) for NG (Table 2). No female subject had a discrepant result for both CT and NG.

When performance was analysed by participant-reported symptomatic status, there was no evidence of a significant difference between symptomatic and asymptomatic patients (p > 0.05). All point estimates were 100% for asymptomatic participants (Table 2). Among symptomatic participants (Table 2), the RPA CT/NG assay's sensitivity was lower: 15 (93.8%) of 16 (95% CI, 69.8–99.8) for male CT FCU and 13 (92.9%) of 14 (95% CI, 66.1–99.8) for female CT SCVS, but specificity and NPV remained high. In addition, all diagnostic accuracy measures for NG detection in both male and female FCU and female CT FCU detection were 100%, regardless of symptomatic status (Supplementary Table S2).

## Discussion

In this diagnostic accuracy evaluation of a prototype ultrarapid isothermal RPA assay for detection of CT and NG, performance (sensitivity, specificity, PPV and NPV) against the reference standard for CT was >94% for all sample types evaluated (male FCU; female FCU and SCVS). Performance for NG was 100% except for SCVS sensitivity and NPV; it was however not possible to assess NG sensitivity and PPV in women confidently because of the low numbers of positive results. The RPA CT/NG also demonstrated excellent technical performance, as no inhibitory results and very few RPA CT/NG assay device errors were observed.

With respect to rapidity, the RPA CT/NG assay's sample preparation, amplification and detection take place in less than 20 minutes, including sample preparation and RPA CT/NG assay run time. A simple-to-use desalting device has been included in newer iterations of the assay, allowing immediate (in seconds) processing of FCU samples before running the assay, although currently it is only appropriate for research laboratory use. The test's rapidity enhances the possibility of implementation as a POCT, enabling test and treat strategies with patients diagnosed and treated in the same clinical visit, and is potentially rapid enough to be incorporated into clinical practice with minimal change to clinical pathways. To date, a major barrier identified for STI POCT implementation has been patient willingness to wait, even for a 90-minute rapid test [11], [18], and the major changes to clinic care pathways necessary to incorporate rapid tests as POCTs as part of SHC consultations [11], [12]. Consequently, CT/NG GeneXpert implementation has enabled a same-day or next-day results service, rather than a POCT test and treat strategy [19], [20], [21]. The RPA CT/NG assay's rapidity, combined with its high performance, therefore has the potential to revolutionize STI diagnosis and management.

Furthermore, the RPA CT/NG assay would be well suited for use in nonlaboratory conditions, both in low- and high-income countries, because of its limited operational requirements. In resource-limited settings, laboratory services for STIs are either not available or are difficult to access (physically and/or financially) and the development and introduction of affordable STI POCTs are part of the strategic direction of the World Health Organization (WHO) [22]. The RPA CT/NG assay fulfils many of the ASSURED criteria, developed by WHO as a benchmark to decide if tests address disease control needs in developing countries [23], and could therefore be an excellent candidate for a true CT/NG POCT in multiple settings.

British Association for Sexual Health and HIV guidelines for NG testing indicate a minimum PPV of 90%; below this, positive results should be confirmed with supplementary testing using a different nucleic acid target from the original test [5]. Although our NG PPV point estimates were all 100%, the lower 95% CI were all <90%. As the RPA CT/NG assay has only a single NG molecular target, it may ultimately have lower specificity and PPV compared to two-target assays, especially if applied to lower prevalence settings. That said, a diagnostic evaluation with large sample size of the two-target GeneXpert by Gaydos et al. [24] also resulted in NG PPVs with lower 95% CIs <90% in all sample types despite the point estimate being >90%, indicating that supplementary testing may be required for both assay types in low prevalence settings.

The RPA CT/NG assay shows promise for both screening of asymptomatics and diagnosis of symptomatics, as we found no significant difference in point estimates by symptomatic status, in accordance with previously reported findings [24]. Furthermore, both SCVS and FCU are possible sample types for women. This is interesting because it has previously been reported that urine is less sensitive than swabs, probably because of lower bacteria load [25]; we did not have data on organism load to explore this finding further. It is also possible that different sample storage (extracted SCVS eluate frozen vs. FCU refrigerated before testing) could have contributed to this finding. Freeze–thaw is unlikely to have impacted on results (one freeze–thaw cycle for FCU before discrepant testing; and a maximum of two freeze–thaw cycles for SCVS, the first for initial testing and the second for discrepant testing), particularly because CT DNA detection by PCR is unaffected by extended (≤2 years) storage [26]. Our results must however be interpreted with caution, as it would have been more appropriate to compare the RPA CT/NG assay FCU results to clinic FCU NAAT results, had these been available, but female FCU is not routinely collected in England. Because of the very high performance of the assay in this evaluation, it is expected that use of the clinic FCU NAAT as the reference standard would have made little difference.

It is known that the discrepant analysis approach used in this study can lead to biases, particularly when the assay under evaluation is also part of the algorithm used to define truly positive and negative results [27]. However, agreement between the initial clinic NAAT and RPA CT/NG assay was very high, with few samples requiring discrepant resolution. Logistical and funding constraints meant an alternative study design (for example, composite reference standard or patient infection status as used for US Food and Drug Administration approval [27]), with a consistent definition for all sample types, was not possible.

The results of our evaluation are promising for the further development of the RPA CT/NG assay, the aims of which should be to: (a) increase CT sensitivity; (b) ensure the PPV remains >90%; (c) ensure usability; and (d) perform larger evaluations to achieve tighter CIs around point estimates, especially for NG. An important addition to this test's development would be validation of extragenital (pharyngeal and rectal) sample types. Extragenital samples are routinely collected for men who have sex with men, with the majority of NG infections in men who have sex with men detected extragenitally [28].

This prototype RPA CT/NG assay had excellent performance characteristics, comparable to currently used NAATs, and fulfils several WHO ASSURED criteria, most notably accuracy, rapidity and thermostability. Its rapidity without loss of performance suggests that once further developed and commercialized, this test could positively affect both clinical practice and public health.

## Transparency Declaration

Funded by TwistDx, St George's, University of London; and by a UK Clinical Research Collaboration, Translational Infection Research Initiative Consortium Grant (grant G0901608). STS reports on behalf of himself and colleagues of the SGUL ADREU grants from UKCRC, other from TwistDx; and RP, PH and NF declare CLRN support to conduct portfolio research during the conduct of the study. STS declares the following relevant financial activities outside the submitted work: other from Atlas Genetics Ltd, other from Alere, other from Cepheid, other from SpeeDx, other from Sekisui, grants from Innovate UK, grants from National Institute for Health Research. MP, OP, MSF and DGB are employees of TwistDx. Advisory board membership is declared by EHE, SF, RP and PH for Becton Dickinson; by RP for Roche, Novartis, GSK, Genoccea and CLJC; and by PH for Hologic and Bayer Consumer Healthcare, outside the submitted work. In addition, MP has a patent EP2029782 issued, a patent EP2426221 issued; and OP has a patent 7 666 598 issued (not owned by the author) and a patent 9 663 820 issued (not owned by the author). The other authors report no conflicts of interest relevant to this article.
