# Supplementary material for: Diagnostic accuracy of a prototype rapid chlamydia and gonorrhoea recombinase polymerase amplification assay: a multicentre cross-sectional preclinical evaluation
Source: Clin Microbiol Infect. 2019 Mar;25(3):380.e1–7. doi: 10.1016/j.cmi.2018.06.003 (PMC6420679; doi:10.1016/j.cmi.2018.06.003)
Supplement: Multimedia component 2 [file mmc2.docx]

| **Supplementary Table S2. Tables of agreement between RPA CT/NG assay and reference standard** | | | | | | | | | | | | | | | | | | | |
| --- | --- | --- | --- | --- | --- | --- | --- | --- | --- | --- | --- | --- | --- | --- | --- | --- | --- | --- | --- |
|  |  | CT | | | | | | | | | NG | | | | | | | | |
|  |  | Males | | | Females | | | | | | Males | | | Females | | | | | |
|  |  | FCU | | | FCU | | | SCVS | | | FCU | | | FCU | | | SCVS | | |
| **A: All participants** | | | | | | | | | | | | | | | | | | | |
|  | Reference standard^a^ | | | | | | | | | | | | | | | | | | |
|  |  | - | + | Total | - | + | Total | - | + | Total | - | + | Total | - | + | Total | - | + | Total |
| RPA CT/NG assay | - | 356 | 2 | 358 | 366 | 0 | 366 | 367 | 1 | 368 | 380 | 0 | 380 | 392 | 0 | 392 | 392 | 1 | 393 |
|  | + | 1 | 33 | 34 | 0 | 29 | 29 | 0 | 27 | 27 | 0 | 12 | 12 | 0 | 3 | 3 | 0 | 2 | 2 |
|  | Total | 357 | 35 | 392 | 366 | 29 | 395 | 367 | 28 | 395 | 380 | 12 | 392 | 392 | 3 | 395 | 392 | 3 | 395 |
| **B: Symptomatic participants^b^** | | | | | | | | | | | | | | | | | | | |
|  | Reference standard^a^ | | | | | | | | | | | | | | | | | | |
|  |  | - | + | Total | - | + | Total | - | + | Total | - | + | Total | - | + | Total | - | + | Total |
| RPA CT/NG assay | - | 125 | 1 | 126 | 172 | 0 | 172 | 172 | 1 | 173 | 132 | 0 | 132 | 184 | 0 | 184 | 184 | 1 | 185 |
|  | + | 1 | 15 | 16 | 0 | 14 | 14 | 0 | 13 | 13 | 0 | 10 | 10 | 0 | 2 | 2 | 0 | 1 | 1 |
|  | Total | 126 | 16 | 142 | 172 | 14 | 186 | 172 | 14 | 186 | 132 | 10 | 142 | 184 | 2 | 186 | 184 | 2 | 186 |
| **C: Asymptomatic participants** | | | | | | | | | | | | | | | | | | | |
|  | Reference standard^a^ | | | | | | | | | | | | | | | | | | |
|  |  | - | + | Total | - | + | Total | - | + | Total | - | + | Total | - | + | Total | - | + | Total |
| RPA CT/NG assay | - | 231 | 0 | 231 | 193 | 0 | 193 | 194 | 0 | 194 | 247 | 0 | 247 | 207 | 0 | 207 | 207 | 0 | 207 |
|  | + | 0 | 18 | 18 | 0 | 15 | 15 | 0 | 14 | 14 | 0 | 2 | 2 | 0 | 1 | 1 | 0 | 1 | 1 |
|  | Total | 231 | 18 | 249 | 193 | 15 | 208 | 194 | 14 | 208 | 247 | 2 | 249 | 207 | 1 | 208 | 207 | 1 | 208 |

FCU, First-catch urine; SCVS, Self-Collected Vulvo-Vaginal Swab; CT, *Chlamydia trachomatis*; NG, *Neisseria gonorrhoeae*.

^a^ Reference standard defined as agreement between at least two of the three tests: clinic nucleic acid amplification test (NAAT), RPA CT/NG assay, Cepheid GeneXpert.

^b^ Male participants considered symptomatic if they reported ≥1 of the following symptoms on the Case Report Form: Discharge (clear or cloudy liquid from the penis); Irritation at the top of the penis; Itching; Needing to pass urine more often than usual; Pain/burning when urinating. Female participants considered symptomatic if they reported ≥1 of the following symptoms on the Case Report Form: Itching; Discharge(clear or cloudy liquid from the vagina); Pain/burning when urinating; Needing to pass urine more frequently; Pain during sex; Bleeding after sex; Bleeding in between periods; Pelvic abdominal pain
