# Supplementary material for: Diagnostic accuracy of a prototype rapid chlamydia and gonorrhoea recombinase polymerase amplification assay: a multicentre cross-sectional preclinical evaluation
Source: Clin Microbiol Infect. 2019 Mar;25(3):380.e1–7. doi: 10.1016/j.cmi.2018.06.003 (PMC6420679; doi:10.1016/j.cmi.2018.06.003)
Supplement: Multimedia component 3 [file mmc3.docx]

| **Supplementary Table S3. Discrepant analysis** | | | | | | | | | | | | | |
| --- | --- | --- | --- | --- | --- | --- | --- | --- | --- | --- | --- | --- | --- |
| A: Male participants, First-catch urine (FCU) | | | | | | | | | | | | | |
| Clinic | **Organism** | RPA CT/NG assay | | Clinic CT/NG NAAT | | GeneXpert | | Resolved result^a^ | | RPA CT/NG assay result interpretation | | | |
|  |  |  | |  | |  | |  | |  | | | |
| 1 | CT | + | | - | | - | | - | | False-positive | | | |
| 1 | CT | - | | + | | + | | + | | False-negative | | | |
| 1 | CT | - | | + | | + | | + | | False-negative | | | |
| **B: Female participants** | | | | | | | | | | | | | |
| Clinic | **Organism** | **First-catch urine (FCU)** | | | | | | | **Self-Collected Vulvo-Vaginal Swab (SCVS)** | | | | |
|  |  | **RPA CT/NG assay** | **Clinic CT/NG NAAT (SCVS)** | | **GeneXpert** | **Resolved result^a^** | **RPA CT/NG assay result interpretation** | | **RPA CT/NG assay** | **Clinic CT/NG NAAT** | **GeneXpert** | **Resolved result^a^** | **RPA CT/NG assay result interpretation** |
| 2 | CT | - | + | | - | - | True-negative | | - | + | - | - | True-negative |
| 2 | CT | - | + | | - | - | True-negative | | + | + | N/A | + | Non-discrepant true-positive |
| 2 | CT | + | - | | + | + | True-positive | | - | - | N/A | - | Non-discrepant true-negative |
| 2 | CT | + | + | | N/A | + | Non-discrepant true-positive | | - | + | - | - | True-negative |
| 2 | CT | + | + | | N/A | + | Non-discrepant true-positive | | - | + | + | + | False-negative |
| 2 | NG | - | + | | - | - | True-negative | | - | + | - | - | True-negative |
| 2 | NG | - | + | | - | - | True-negative | | - | + | - | - | True-negative |
| 2 | NG | - | + | | - | - | True-negative | | - | + | - | - | True-negative |
| 2 | NG | + | + | | N/A | + | Non-discrepant true-positive | | - | + | + | + | False-negative |

^a^ Resolved result defined by 2 of the 3 results (clinic NAAT, RPA CT/NG assay, Cepheid GeneXpert) in agreement
